# Origins of multicellular evolvability in snowflake yeast

**DOI:** 10.1038/ncomms7102

**Published:** 2015-01-20

**Authors:** William C. Ratcliff, Johnathon D. Fankhauser, David W. Rogers, Duncan Greig, Michael Travisano

**Affiliations:** 1 School of Biology, Georgia Institute of Technology, Atlanta, Georgia 30332-0230, USA; 2 Plant Biology, University of Minnesota, St Paul, Minnesota 55108, USA; 3 Max Planck Institute for Evolutionary Biology, 24306 Plön, Germany; 4 Department of Genetics, Evolution, and Environment, University College London, London WC1N 6BT, UK; 5 Department of Ecology, Evolution and Behavior, University of Minnesota, St Paul, Minnesota 55108, USA; 6 The BioTechnology Institute, University of Minnesota, St Paul, Minnesota 55108, USA

## Abstract

Complex life has arisen through a series of ‘major transitions’ in which collectives of formerly autonomous individuals evolve into a single, integrated organism. A key step in this process is the origin of higher-level evolvability, but little is known about how higher-level entities originate and gain the capacity to evolve as an individual. Here we report a single mutation that not only creates a new level of biological organization, but also potentiates higher-level evolvability. Disrupting the transcription factor *ACE2* in *Saccharomyces cerevisiae* prevents mother–daughter cell separation, generating multicellular ‘snowflake’ yeast. Snowflake yeast develop through deterministic rules that produce geometrically defined clusters that preclude genetic conflict and display a high broad-sense heritability for multicellular traits; as a result they are preadapted to multicellular adaptation. This work demonstrates that simple microevolutionary changes can have profound macroevolutionary consequences, and suggests that the formation of clonally developing clusters may often be the first step to multicellularity.

## Abstract (web summary)

The first steps in the transition to multicellularity remain poorly understood. Here, the authors demonstrate that disrupting a single gene in yeast results in multicellular clusters that develop clonally and possess a high degree of multicellular heritability, predisposing them to multicellular adaptation.

## Graphical abstract

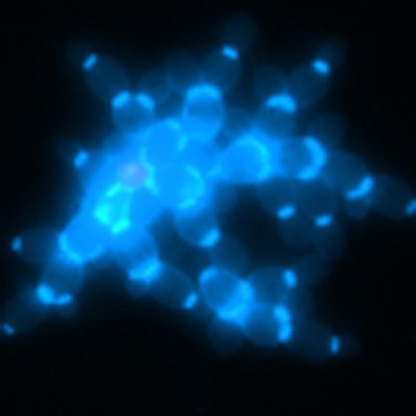

One of the most conspicuous features of Earth’s organisms is their complexity. In their paradigm-defining synthesis, John Maynard Smith and Eörs Szathmáry identified key steps (termed ‘major transitions’) through which complexity evolves^1^. The multilevel selection hypothesis for major transitions posits a two-step process: first, a solitary ancestor evolves to form collectives^2^, then selection shifts to the collective level^1^,^3^,^4^. Biological complexity arises as a result of adaptation in the new, higher-level organismal unit^1^,^5^,^6^,^7^. The multilevel selection hypothesis has strong historical and theoretical support for many of the major transitions in evolution (for example, origins of cells, chromosomes, eukaryotes, multicellularity and eusocial superorganismality^1^,^8^,^9^,^10^), but understanding how higher-level entities originate and gain the ability evolve as Darwinian Individuals remains a challenge^11^,^12^.

The conditions required for higher-level adaptation are stringent^13^. First, collectives must be capable of reproducing^6^,^7^,^12^. Second, collectives must have the properties required for Darwinian evolution, namely they must vary in their collective-level traits, this variation must be heritable, and these collective-level traits must affect fitness^9^,^12^,^13^,^14^. Finally, internal conflicts must be minimized. Collective-level adaptations can easily be undermined by within-collective (lower-level) evolution^15^,^16^,^17^,^18^,^19^, particularly if higher-level adaptations reduce the fitness of lower-level parts (for example, cellular division of labour). Collectives that undergo a bottleneck during reproduction limit lower-level genetic diversity, reducing the potential for conflict between the fitness of the collective and its constituents^1^,^16^,^20^,^21^,^22^,^23^,^24^,^25^,^26^. How do incipient multicellular organisms meet these criteria and gain the capacity to evolve as Darwinian individuals?

We can glean several clues from life’s successful and failed transitions in individuality. Collectives of like individuals (‘fraternal’ transitions)^27^ are thought to be important for the evolution of chromosomes from independent replicators, multicellular organisms from solitary cells and eusocial ‘super organisms’ from asocial multicellular ancestors. These collectives faced the classic problems of group selection, namely that cluster-level adaptation requires that the strength of among-collective selection exceed the strength of within-collective selection^28^. Heritable diversity among lower-level units within collectives is a key factor determining the relative strength of lower- versus higher-level selection^29^. Clonal collectives align the fitness interests of lower-level units, and as a result the primary way for a lower-level unit (for example, a cell) to increase its fitness is by enhancing the collective’s fitness (for example, a multicellular organism). Most multicellular- and super-organisms have solved this problem by producing propagules that develop through a unicellular genetic bottleneck, limiting migration of lower-level units between collectives^1^,^16^,^21^,^22^,^23^,^25^,^30^,^31^. The single-cell bottleneck and subsequent clonal development is thus a key trait facilitating the evolution of higher-level complexity in fraternal transitions. Two widely studied social organisms, the slime mold *Dictylostelium discoideium* and bacterium *Myxococcus xanthus*, appear stuck in the transition to multicellularity, despite ample time to evolve multicellular complexity (>400 Myr ago for the Dictyostelid cellular slime molds^32^ and >650 Myr ago for the myxobacteria^33^). While both organisms possess multicellular life histories that include cellular division of labour, neither life cycle includes a single-cell bottleneck, and genetic conflict is rampant^18^,^34^,^35^. This conflict can select for adaptations that limit the diversity of cells within collectives (for example, policing^36^,^37^, greenbeards^38^), but these mechanisms are not as effective or evolutionarily durable as the single-cell bottleneck.

Collectives of unlike individuals (‘egalitarian’ transitions)^27^ are thought to be critical for the evolution of cells from populations of replicating molecules, chromosomes from unlinked replicators and eukaryotes from a symbiotic pair. Multispecies collectives are common in nature, ranging in size and complexity from communities to pairs of species interacting synergistically (for example, cross-feeding microbial consortia). Multispecies collectives can possess a substantial fitness advantage relative to solitary competitors^39^,^40^,^41^, and thus provide a rich substrate for selection among collectives to facilitate higher-level adaptation. Yet few multispecies collectives have made the transition to a higher level of individuality. Multispecies collectives encounter all of the challenges faced by uni-species collectives (see above), as well as the added difficulty of regenerating a collective from two or more distinct genetic backgrounds. In the absence of a developmental mechanism that ensure partner fidelity across multiple generations of the collective (for example, co-dispersal^42^,^43^, vertical transmission^44^ or partner choice^45^), the heritability of collective-level traits collective is limited. The importance of transmission mode is illustrated by differences in symbiosis: only vertically transmitted symbionts have become a part of a new, higher-level organism (for example, the multiple origins of plastids^46^). In contrast, horizontally transmitted symbionts (for example, legumes and *Rhizobium*, bobtail squid and *Vibrio*) have failed to make the transition from symbiont to organelle. Taken together, it is clear that collectives that successfully transit to a higher level of individuality possess heritable multicellular traits that selection may act on, and exhibit little within-group conflict.

Here we use the transition from uni- to multicellularity as a model to investigate the origin of higher-level evolvability. We previously evolved multicellularity in the yeast *Saccharomyces cerevisiae.* Starting with a single diploid clone of strain Y55 (a unicellular yeast), we selected for rapid settling through liquid media in 10 replicate populations. Within 60 daily transfers, multicellular ‘snowflake’ yeast evolved in all 10 populations, displacing their unicellular ancestors. Snowflake yeast result from daughter cells remaining attached to their parent cells after mitosis. Snowflake yeast display a key emergent property: as clusters grow larger, tension among cells increases until it exceeds the tensile strength of a cell–cell connection, resulting in the release of a multicellular propagule^5^. Once clusters have evolved, they readily become a unit of selection, as whole clusters either settle rapidly enough to survive, or fail to do so and perish. As a result of this shift to cluster-level selection, we observe extensive cluster-level adaptation, including the evolution of larger size, elevated apoptosis and more spherical, hydrodynamic clusters^5^,^47^. While the evolution of larger clusters reduces their number in the population, our cluster-level effective population size remains large, minimizing the role of genetic drift. Even in one of our largest cluster-forming strains from 60 days, the effective population size (*N*_e_) of clusters is still 9.4 × 10^5^ (calculated as the harmonic mean of population size variation over each day).

We follow first principles in examining the origin of multicellular evolvability in our yeast model system. We first characterize the genetic basis of multicellularity in snowflake yeast and then describe their three-dimensional developmental pattern mathematically. We next determine how this growth form affects the within-collective genetic variation, and examine its implications for the evolution of within-cluster genetic conflict and multicellular heritability. We find that the snowflake yeast developmental pattern imbues clusters with a high degree of multicellular evolvability, demonstrating that seemingly simple molecular changes can have profound macroevolutionary consequences.

## Results

### Genetic basis of the snowflake yeast developmental pattern

**Figure 1:**
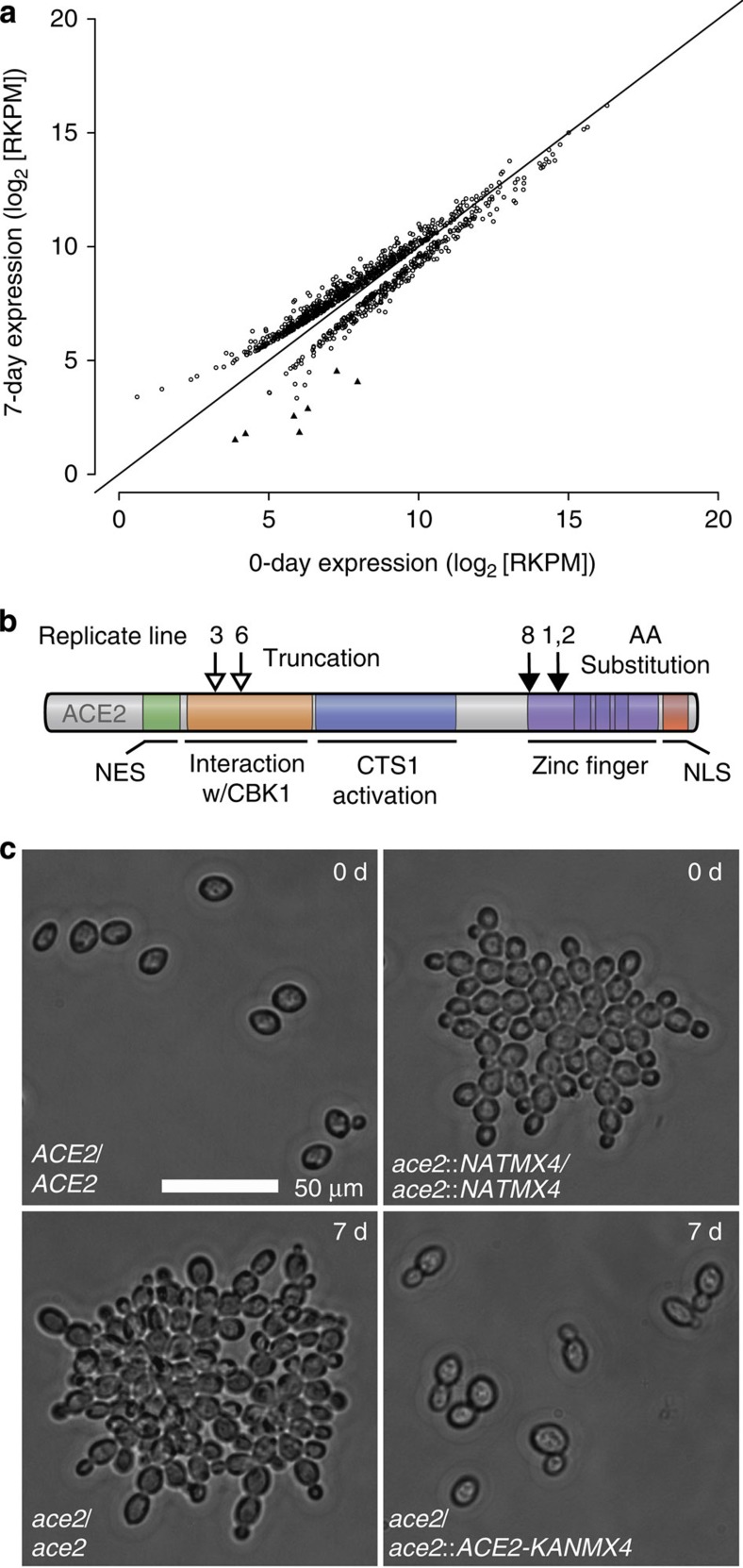
Genetic basis of multicellularity. (**a**) 1,035 genes were differentially expressed between the unicellular ancestor and an early (7-day) snowflake yeast. Of the 10 most downregulated genes, seven are regulated by the transcription factor *ACE2* (triangles). (**b**) Non-synonymous mutations were found in the *ACE2* of 5/10 lineages (open arrows designate nonsense mutations, closed arrows designate missense mutations). (**c**) Knocking out *ACE2* in the unicellular ancestor (upper right) results in snowflake yeast, while functionally complementing the 7-day strain (lower right) restores unicellularity.

Comparing the gene expression between the unicellular Y55 ancestor and an early (7-day) snowflake yeast, we found that 1,035 genes were significantly differentially expressed 8 h after transfer. Of these, only 143 genes differed by more than twofold. Of the 10 most downregulated genes (Table 1), seven (*CTS1*, *DSE4*, *DSE2*, *SUN4*, *DSE1*, *SCW11* and *AMN1*) are regulated by the *trans*-acting transcription factor *ACE2* (marked with triangles in Fig. 1a; Tables 1, 2, 3), suggesting that the native function of *ACE2* is disrupted in early snowflake yeast. These seven most downregulated genes are involved in daughter cell separation, many acting directly to degrade the bud neck septum^48^,^49^,^50^, and prior work has shown that *ACE2* knockouts form cellular clusters^48^,^51^,^52^. We next sequenced *ACE2* from 10 independently evolved lineages of snowflake yeast (populations 1–10 from Ratcliff *et al*.^5^). Non-synonymous mutations were detected in five populations, either causing protein truncation (populations 3 and 6) or amino-acid substitution in (or adjacent to) *ACE2*’s zinc finger-binding domain (populations 1, 2, 8; Fig. 1b and Table 2). In all cases, both *ACE2* alleles in the diploid yeast were identical, suggesting that the mutant *ACE2* allele was made homozygous by gene conversion, a common occurrence in yeast^53^. These mutations likely lead to non (or less)-functional ACE2p. We confirmed that a loss of *ACE2* functionality would develop the snowflake phenotype by constructing a diploid *ace2::NATMX4/ace2::NATMX4* knockout in the unicellular ancestor (Fig. 1c). Further, we found that complementation of an experimentally evolved snowflake yeast (population 1, codon 645^val » asp^) with a single copy of functional *ACE2* linked to *KANMX4* resulted in reversion to unicellularity (Fig. 1d). The snowflake yeast phenotype can therefore be produced by the disruption of a single gene.

### Modelling the snowflake yeast developmental pattern

**Figure 2:**
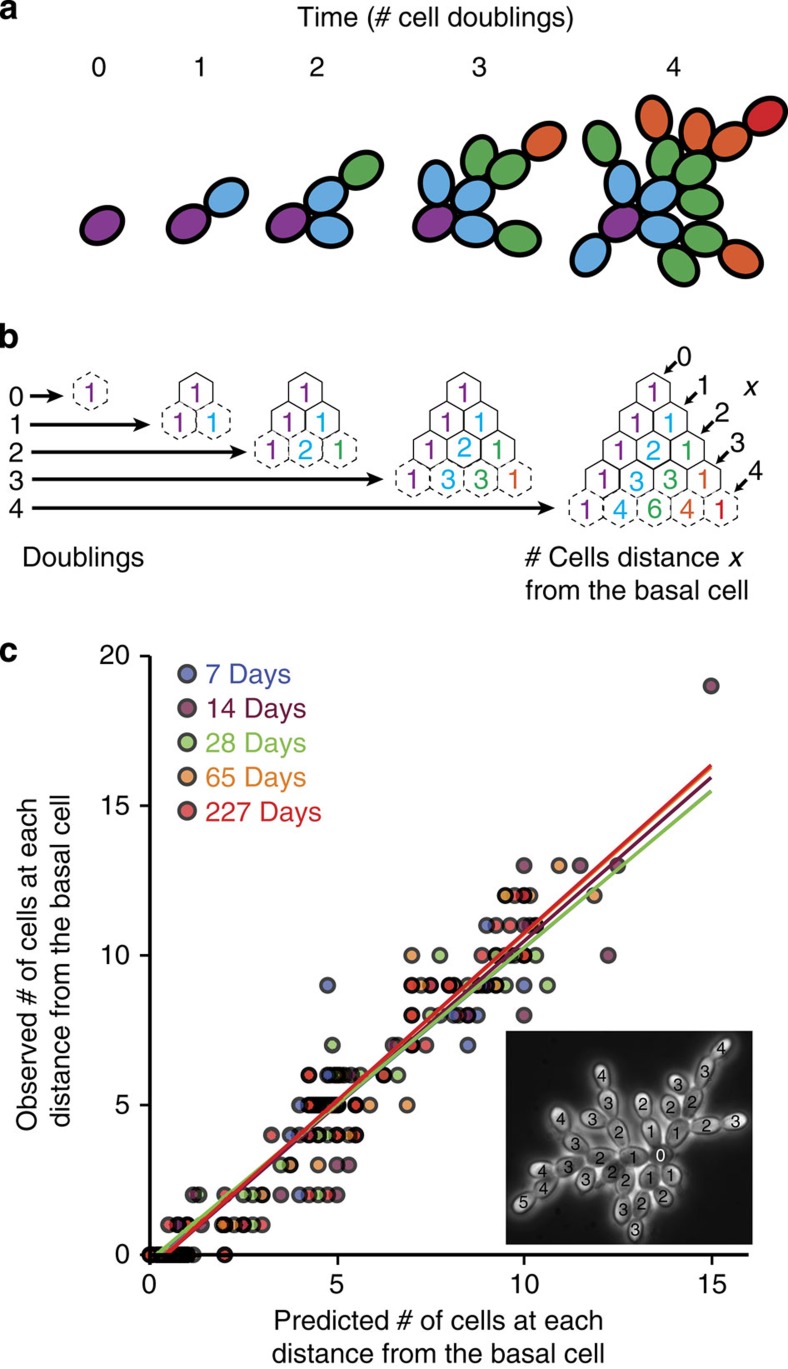
Modelling the snowflake yeast body plan. (**a**) Cluster growth from a single cell, with each distance from the basal cell (purple) labelled a different colour. (**b**) Pascal’s triangle describes the number of cells distance *x* from the basal cell. (**c**) The model accurately predicts the growth form of snowflake yeast. Despite a 45% increase in settling speed over 227 days of evolution^21^, we find no differences in the growth form of any of our snowflake yeast isolates. Inset: a cluster with each cell’s position from the basal labeled.

Snowflake yeast clusters grow by daughter cell adhesion to maternal cells (Fig. 2a). This results in a mathematically tractable growth form where, if cells reproduce at the same rate, the number of cells distance *x* from the basal cell follows Pascal’s triangle (Fig. 2b). The number of cells distance *x* from the basal cell *c*_*d*_(*x*) can be calculated after *d* doublings with the binomial expression 
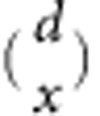
. In practice, all cells do not divide synchronously, so we modified the above binomial to include a partial doubling: the reproduction of fraction *s* cells. We therefore can describe the structure of a snowflake yeast cluster of any size by predicting the number of cells distance *x* from the basal cell (see Supplementary Methods for a complete derivation of the model):


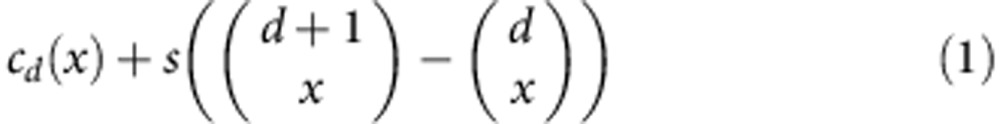


To assess our model’s description of snowflake yeast growth form, we compared the predicted number of cells of each distance from the basal cell (0, 1, 2, 3…*x*) with experimentally obtained counts. To generate this prediction, we calculated the number of complete doublings from the basal cell, *d*, as log_2_(*cell number*), rounded down. We also calculated *s*, the fraction of cells within the cluster that reproduced from the last complete doubling, as described above. Inputting the values of *d* and *s* into our model, we obtained the expected number of cells at each distance *x* from the basal cell. We then compared the actual number of cells at each distance from the basal cell, determined empirically (Fig. 2c), to the number predicted by our model. Our model accurately described the growth form of snowflake yeast clusters (F_9,311_=574.7, *P*<0.0001, main effect of predicted number of cells per generation in an analysis of covariance with observed number of cells per generation as the response variable and yeast strain as the cofactor, overall *r*^*2*^=0.94. The main effect of yeast strain and the interaction with the cofactor were not significant Fig. 2d).

The growth form of snowflake yeast conforms to what we would expect if newly produced cells remain attached to their parent cell, and it takes each cell a similar amount of time to reproduce. Our analysis implies that the snowflake yeast growth form does not change during the course of 227 days of experimental evolution (~1,135 generations), despite substantial changes in cell size, cluster size and settling velocity^47^. Using ideal snowflake yeast clusters generated from the model above, we examine key evolutionary consequences of this developmental pattern.

### Propagules always experience a unicellular genetic bottleneck

**Figure 3:**
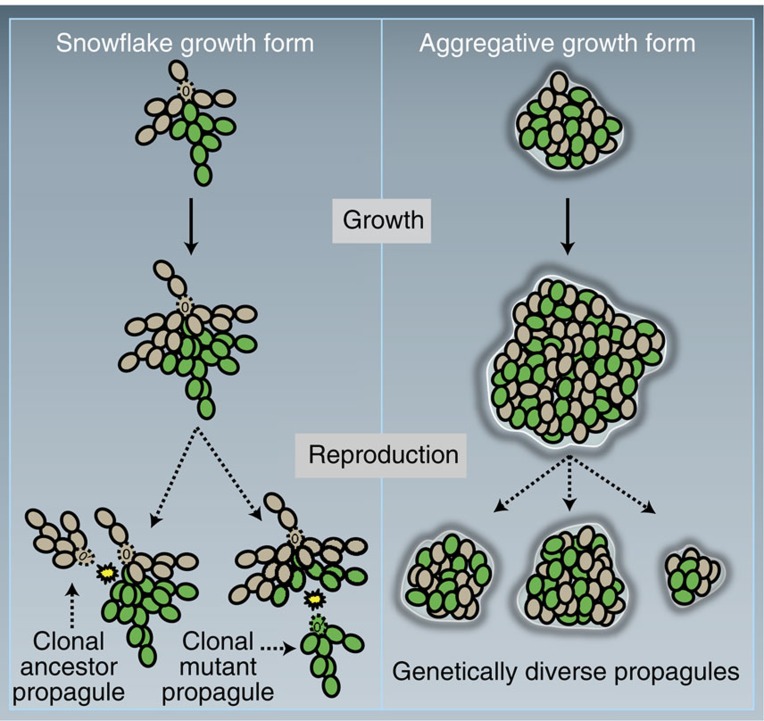
Snowflake versus aggregate life cycles. Segregation of *de novo* mutations into mutant-only clusters is inevitable in snowflake yeast, but unlikely in aggregates. Shown are the chimeric clusters containing wild-type (tan) and mutant (green) lineages. Snowflake clusters reproduce by severing a single cell–cell connection (cells with a dashed outline marked 0′), resulting in a unicellular genetic bottleneck. Only two types of propagule can be generated: those with an ancestral cell at the bottleneck (resulting in a purely ancestral propagule) or those with a mutant cell at the bottleneck (resulting in a purely mutant propagule). In contrast, the aggregative life cycle has no bottleneck. Instead, propagules are formed by randomly selecting cells from within the cluster.

Each time a propagule separates from a parent cluster, it goes through a single-cell genetic bottleneck, even if the propagule is multicellular. Consider the separation of any two connected cells in a snowflake yeast cluster. This will cause fragmentation and production of a new cluster (Fig. 3a). Due to the way that snowflake yeast grow, one of the two resulting clusters will always be one of the ‘branches’ of cells from the parent cluster (except in the case where it is a single cell), and this branch will always contain a single cell at its base (denoted by a dashed outline and 0’ annotation in Fig. 3a). All other cells in the propagule are clonal descendants of this basal cell. Thus all propagules experience single-cell genetic bottlenecks, despite multicellular propagation. This mode of reproduction greatly limits within-cluster genetic diversity and acts as a conflict mediator^54^, facilitating multicellular adaptation^1^,^16^,^20^,^21^,^22^,^23^,^24^,^25^,^26^.

### Snowflake yeast rapidly purge within-cluster genetic variation

**Figure 4:**
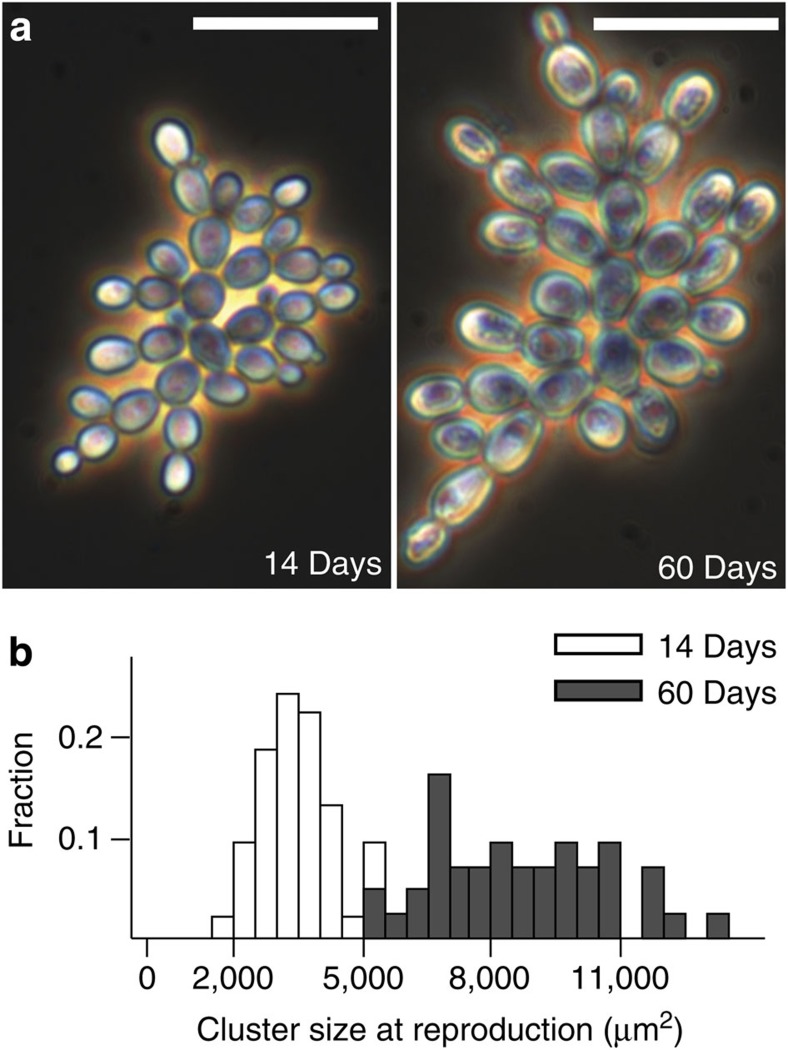
Change in cell-level traits affects cluster-level traits. (**a**) By 60 days, cells have evolved to be 2.2-fold larger than an early snowflake yeast from the same population (14 days). This increases cluster size and settling rate. Both clusters are shown at the same magnification. Scale bars, 50 μm. (**b**) These large-celled yeast have evolved to produce propagules when 2.5-fold larger than the 14-day strain, on average. The broad-sense heritability of this life history trait is 0.84.

Selection in our experimental populations readily acts on the properties of whole clusters, such as settling speed. Consider the evolution of larger cells, a trait that evolves in our experimental populations (Fig. 4a) and is predicted to increase cluster size and settling speed by ~45% (ref. 47). Cluster-level selection favours the groups composed entirely of large-celled yeast, but cluster-level selection alone cannot easily increase the frequency of a large-cell mutation when the mutant is rare. This is because the benefit of faster settling is shared with a greater number of wild-type (small) cells in the same cluster. How then does selection favour initially rare mutations that confer only a cluster-level fitness benefit?

**Figure 5:**
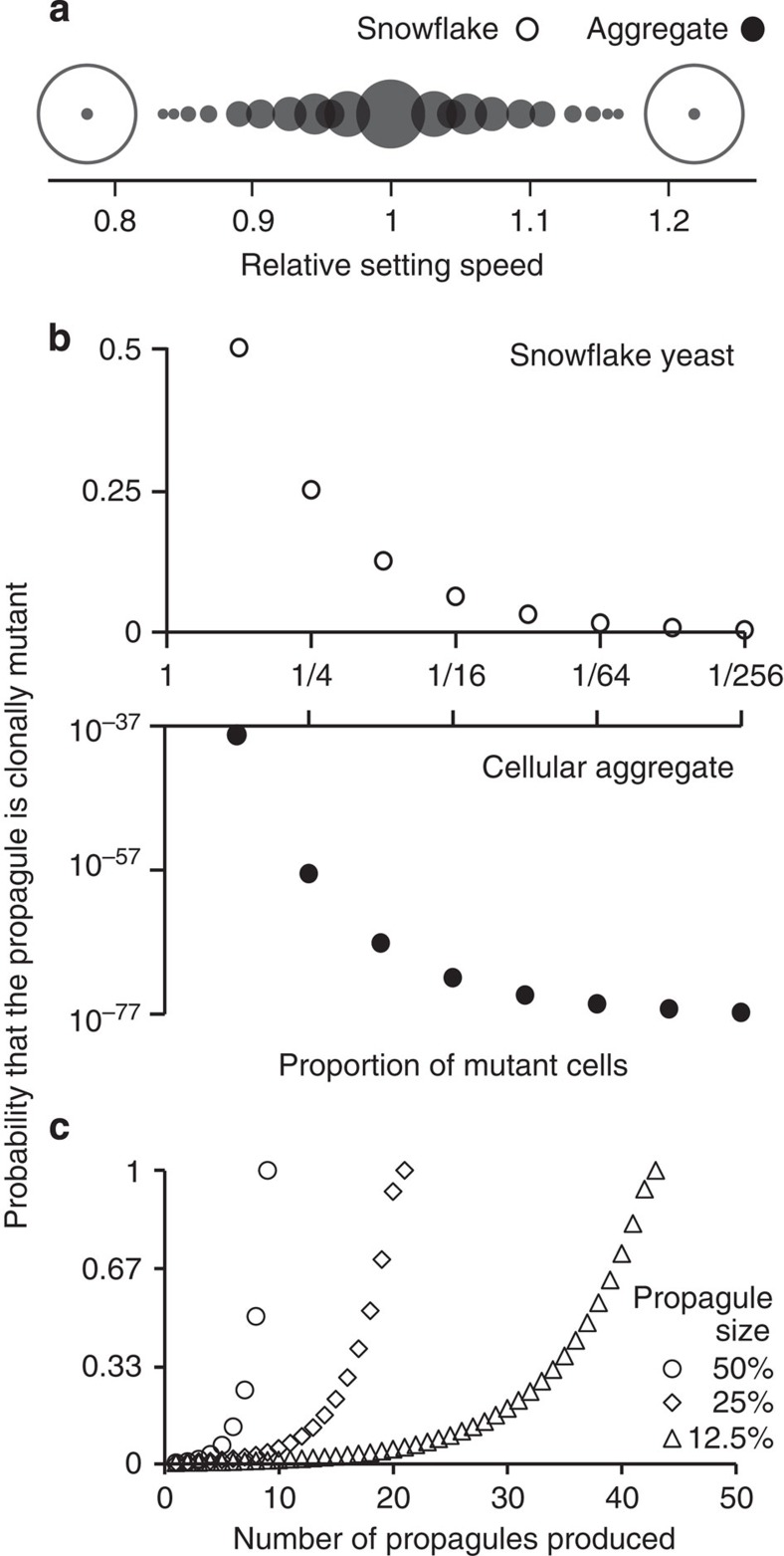
The snowflake yeast body plan ensures genetic segregation. (**a**) We model the settling rate of propagules produced by 16-celled snowflake yeast clusters (open circles) or cellular aggregates (filled circles) that contain 50% small and large cells. Snowflake yeast produce offspring in which small- and large-cell alleles are completely segregated into different clusters (resulting in either slow or fast settling speed), while most aggregates contain a mixture of both cell types. (**b**) We model the probability that a propagule produced by a 256-cell cluster containing both wild-type cells and a mutant lineage will contain only mutant cells as a function of mutant frequency in the cluster. Snowflake yeast clusters have a far higher probability of producing mutant-only propagules than aggregate clusters. (**c**) Rare mutants in 256-cell snowflake yeast clusters initially have a low probability of forming their own propagules, but mutant-only propagules are eventually assured.

Cluster-level selection can easily favour cellular traits that provide no within-group fitness advantage (such as large cell size) when genetic variation is effectively partitioned between groups. To examine how the dynamics of cluster developmental mode affect the genetic composition of propagules, we model ideal snowflake yeast clusters and contrast this with cellular aggregates. Cellular aggregates, which can be thought of as biofilm-like structures (similar to yeast flocs), do not necessarily undergo a genetic bottleneck when producing propagules. We assume propagules are up to half of the parent cluster’s size, and that the mutation leading to larger cells only occurs once within a cluster, after which it is passed on to daughter cells within the cluster. Consider a 16-celled cluster containing 50% small and large cells. Snowflake yeast, whose propagules pass through a unicellular genetic bottleneck, completely segregate genetic variation among offspring, while only 0.6% of the propagules produced by aggregative clusters contain just the large-celled mutant (Fig. 5a). We arrive at this result as follows: in this snowflake yeast cluster, all cell–cell connections that are broken must be between either two mutant cells (yielding a mutant-only propagule), a mutant cell and a wild-type cell (yielding two equal-sized uniclonal mutant and wild-type clusters) or between two wild-type cells (yielding a wild-type-only propagule). Within aggregative clusters, *n* cells (up to half the size of the cluster) are chosen randomly for inclusion in propagules. The number of ways the aggregative cluster can produce 100% large-celled propagules is 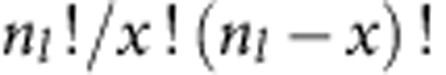, where *n*_l_ is the number of large cells in the cluster, and *x* is propagule size. Similarly, the probability of getting 100% small cells is 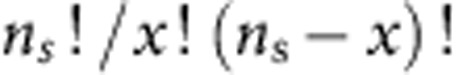, where *n*_s_ is the number of small cells in the cluster. Finally, the total number of non-clonal propagules can be determined by 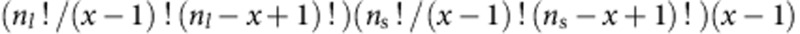. We generate the result in Fig. 5a by dividing the total number of clonal propagules from the total number of non-clonal propagules for offspring size 1–8.

The model presented in Fig. 5a considered mutants at high frequency. Rare mutants provide an even stronger advantage for snowflake yeast. A mutant-only propagule will be produced when fragmentation occurs between either the mutant and a wild-type cell, or two mutant cells. When all breakages between cells are equally likely, this is *n*_m_/(*n*_tot_−1), where *n*_m_ and *n*_tot_ are the number of mutant and total cells in the cluster, respectively. With the snowflake yeast body plan, the probability of producing a uniclonal mutant propagule is therefore approximately the same as the mutant frequency in the cluster (Fig. 5b). For aggregate clusters, the probability that a randomly produced propagule from an aggregate will be mutant only is:


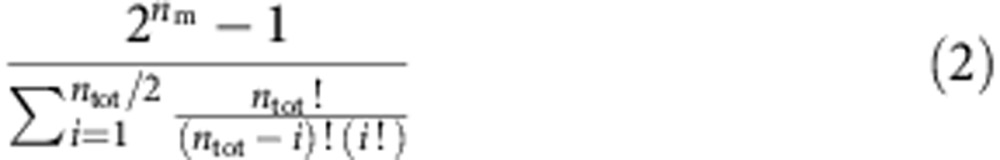


This is simply the fraction of total unique propagules of size 1 to *n*_tot_/2 (denominator) that will contain only mutant cells (numerator). The probability of producing a mutant-only propagule is negligible (~10^−39^ for a 256-cell cluster containing 50% mutant cells, Fig. 5b), and declines exponentially with reduced mutant frequency. As a result, segregation of the mutant into its own clusters will be extremely rare, limiting the capacity for selection to act on the multicellular phenotype of individual mutations (a limitation similar to that imposed by ‘blending’ modes of inheritance^55^).

Rare mutant cells have little chance of founding a uniclonal propagule, even in snowflake yeast (Fig. 5b). However, when wild-type-only propagules are produced, the frequency of mutant cells in the snowflake parent cluster is increased. As a result, the probability that the next propagule to be produced will be mutant-only (*p*_m_) increases with each wild-type-only propagule that is produced (Fig. 5c),


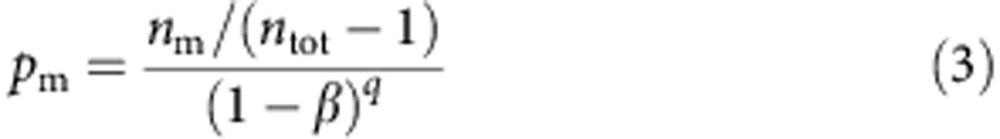


where *β* is the size of each propagule relative to the whole cluster before division and *q* is the number of wild-type-only propagules produced. The snowflake body plan thus ensures that all mutants will eventually form uniclonal propagules, regardless of their initial frequency in a cluster (Fig. 5c), allowing the cluster-level phenotypic effects of all *de novo* mutations to be subject to selection.

### Heritability of a key multicellular life history trait

We measured the heritability of a key multicellular life history trait in snowflake yeast, cluster size at reproduction, for strains isolated after either 14 or 60 transfers from the same population (shown in Fig. 4a). For each strain, we performed seven life history analyses, measuring the size of parent and offspring clusters at reproduction over ~12 h of growth (Fig. 4b). The broad-sense heritability of this trait was 0.84, which is extraordinarily high even by the standards of extant clonally reproducing multicellular organisms. For example, the *H*^2^ of six life history traits of the colonial ascidian *Botryllus schlosser* range from 0.2 to 0.75 (ref. 56) and the average *H*^2^ of five life history traits of *Daphnia obtuse* ranged from 0.35 to 0.47, with a maximum of 0.83 (ref. 57). The snowflake developmental pattern creates clusters with high heritability by minimizing phenotypic variation among clusters of the same clone.

## Discussion

Snowflake yeast evolve in as little as 7 days from a unicellular ancestor^47^. Snowflake yeast result from a single mutation (loss of the transcription factor *ACE2*, Fig. 1), which causes a simple change in the growth form of their unicellular ancestor: cells produced mitotically remain attached to their mother cells rather than separating. The snowflake developmental pattern (Fig. 2) creates clusters predisposed to multicellular adaptation. Clusters readily reproduce, creating offspring by fragmentation when tension among cells in the cluster exceeds the tensile strength of cellular adhesion^5^. Every time a new propagule separates from its parent cluster, it goes through a unicellular genetic bottleneck (Fig. 3). As a result, snowflake yeast clusters are typically uniclonal, nullifying the potential for within-group genetic conflict to erode multicellular complexity^1^,^16^,^20^,^21^,^22^,^23^,^24^,^25^,^26^. This does not require any anticheating adaptations (for examples, see refs 37, 58), rather it is a geometric consequence of the snowflake yeast body plan. In addition to limiting genetic conflict, segregation of genetic variation among clusters also increases the multicellular evolvability by exposing the multicellular phenotype of novel mutations to selection (Fig. 4). Clusters of the same clone are physiologically similar (for example, similar cell size and shape, cell–cell adhesive strength, frequency of apoptosis, identical growth form and so on), minimizing within-clone variability and increasing the heritability of multicellular traits (Fig. 5). This means that cluster-level selection can cause rapid evolutionary change, and can act on mutations with relatively small multicellular-level phenotypic effects. These experiments demonstrate that a single genetic change not only can create a new level of biological organization, but can also facilitate the transition to higher-level individuality by potentiating higher-level evolvability.

The evolution of multicellularity reveals how microevolutionary processes of selection and adaptation can cause macroevolutionary phenotypic changes. Newly evolved multicellular individuals are not yet optimized by prior selection; as a result, mutations having dramatic phenotypic effects are more likely to be beneficial than previously expected^59^,^60^. This observation helps to reconcile a long-standing debate in biology on the apparent incommensurability between macro- and microevolution and their relative importance in biological diversity^61^,^62^,^63^. Further, the specific mode of multicellularity evolved in our experiments reveals how novel, higher-level evolvability can readily arise through evolution. Snowflake multicellularity provides a key evolutionary benefit, unicellular genetic bottlenecks, which should be achievable by any organism in which clonal daughters attach just to their mothers. Once cluster growth meets volumetric constraints (which occur even with optimal packing of cells in a sphere^64^), further growth results in cell–cell scission, producing a propagule in which the oldest cell in the cluster is the genetic bottleneck. As a result, even simple clusters may be primed for higher-level adaptation. The exceptional evolvability of multicellular snowflake yeast arises as a *consequence* of the evolution of multicellularity, rather than selection for evolvability itself, sidestepping potential hurdles for its selection^11^,^12^.

## Methods

### RNA-seq

To prepare cells for RNA extraction, all yeast were inoculated 1:100 into 10 ml Yeast Peptone Dextrose medium (YPD; per l: 20 g glucose, 20 g peptone, 10 g yeast extract), grown for 24 h, then diluted 1:100 into fresh YPD and grown for 8 h. Growth conditions were as described in ref. 5. Total RNA was extracted with the Qiagen RNeasy mini kit and then libraries were constructed using the Illumina TruSeq kit. RNA was quantified via fluorimetry using the RiboGreen assay, and RNA integrity was assessed using capillary electrophoresis on the Agilent BioAnalyzer 2100. Libraries were created, size selected and validated by staff at the University of Minnesota Genomics Center (UMGC). Using the Illumina HiSeq 2000, ~10 million 50 bp PE reads were collected per sample. Reads were mapped to the Y55 genome (Sanger) to identify single-nucleotide polymorphisms in coding regions, and the S288C genome for expression analysis (mapping done in CLC Genomics Workbench v. 6.1). Gene expression was measured as reads mapped per kilobase of exon per million reads mapped (RPKM)^65^. RKPM values were then log_2_ transformed for graphical display in R. Gene expression was considered significantly different between unicellular and 7-day multicellular strains when overall false discovery rate-corrected *P* values <0.05 (ref. 66). Genes with very low expression levels (RPKM<1) were excluded. Gene ontology enrichment analysis of differentially expressed genes was conducted using GeneCodis3 (refs 67, 68).

### Sequencing *ACE2*

Genomic DNA was extracted using the Zymo YeaStar kit from one snowflake yeast isolate derived from each of the 10 replicate populations described in Ratcliff *et al*.^5^ *ACE2* (414 bp upstream and 433 bp downstream of the CDS, primers available on request) was PCR amplified and Sanger sequenced at the UMGC.

### Transformation

To test the hypothesis that the loss of *ACE2* was sufficient to generate the snowflake phenotype, we knocked out *ACE2* in our unicellular ancestor (strain Y55) through replacement of the *ACE2* ORF with *NATMX4* using the LiAc/SS-DNA/PEG method of transformation^69^*. ace2::NATMX4/ace2::NATMX4* homozygotes were generated by the autodiploidization of single spores dissected from tetrads formed by heterozygous transformants; tetrads were dissected onto YPD and then *ace2ΔNATMX4/ace2ΔNATMX4* diploids were selected by replica plating to YPD with 100 mg l^−1^ nourseothricin (obtained from Werner Bioagents, Jena, Germany). The genotype was confirmed by PCR, as well as ensuring that all four spores of the selected isolate were nourseothricin resistant. To confirm that the loss of *ACE2* functionality is responsible for the snowflake yeast growth form in our experimentally evolved yeast, we functionally complemented an isolate obtained after 7 days of selection (population 1) with the ancestral *ACE2* allele. To accomplish this, we first inserted a *KANMX4* cassette downstream of the Y55 *ACE2* 3′ UTR (position 404446 on chromosome 7). We then replaced a single copy of the 7-day snowflake yeast with this *ACE2-KANMX* fusion via the LiAc/SS-DNA/PEG method of transformation, plating cells on YPD with 200 mg G418/L.

### Testing the model of the snowflake yeast body plan

To assess our model’s description of snowflake yeast growth form, we compared the predicted number of cells of each distance from the basal cell (0, 1, 2, 3…*x*) with experimentally obtained counts. To generate this prediction, we calculated the number of complete doublings from the basal cell, *d*, as log_2_(cell number), rounded down. We also calculated *s*, the fraction of cells within the cluster that reproduced from the last complete doubling, as described above. Inputting values of *d* and *s* into our model, we generated the expected number of cells at each distance *x* from the basal cell. We then compared the actual number of cells at each distance from the basal cell, determined empirically (for example, Fig. 2c), with the number predicted by our model.

We tested predictions of our model against empirically obtained branching pattern data from 7–10 clusters of five strains of snowflake yeast, isolated after either 7, 14, 28, 65 or 227 days of experimental evolution. On days 28 and 65, the intensity of settling selection was increased to continue selecting for larger cluster size (see ref. 47) for a complete description of the experiment). Clusters were grown from single cells that were obtained via enzymatic digestion with lyticase and β-glucuronidase as described in ref. 5. Clusters were grown in YPD medium, at 30 °C with 250 r.p.m. shaking, and then imaged once after 6 h of growth. All microscopy was performed on an Olympus IX70 inverted microscope with a Scion CFW-1310C camera. We empirically determined each cell’s distance from the basal (seed) cell. See the inset of Fig. 2c for an example of a cluster with this distance annotated.

### Calculating broad-sense heritability of cluster size at reproduction

For each strain, we performed seven life history analyses, measuring the size of parent and offspring clusters at reproduction over ~12 h of growth. Following the procedure of van Kleunen *et al*.^70^, *H*^2^ was calculated using REML analysis of variance to parse the variance components (VC) of genotype and replicate LH analysis effects: *H*^2^=*VC*_genotype_/(*VC*_genotype_+*VC*_LH analysis replicate_+*VC*_residual_), with LH analysis replicate (a random effect) nested in strain (a random effect).

### Quantifying cluster size at reproduction

Time-lapse movies were conducted following the protocol described in ref. 5. In brief, individual clusters from a single-strain isolate (put through three rounds of single-colony isolation) were inoculated into 0.5-μl droplets in Nunc Lab-Tek II chambered cover glass slides. To limit evaporation, 10 μl of water was placed on the walls of the chamber and the chamber sealed with clear tape. Illumination was kept to a minimum to avoid heating the sample. Images were acquired every minute for 16 h. We determined the cluster size at reproduction by measuring the two-dimensional (top–down) footprint of the cluster one frame before the propagule separated in ImageJ.

### Statistical analysis

All statistical tests were conducted in JMP 9.0. Assumptions of parametric tests were checked before use.

## Author contributions

W.C.R. and M.T. conceived of the project. J.D.F. performed and analysed the RNA/DNA sequencing. D.W.R. and D.G. helped W.C.R. plan and execute the genetic transformations. W.C.R. conducted the remaining experiments, analysed their data, performed the modelling and wrote the first draft of the paper (all authors, especially M.T., provided constructive feedback).

## Additional information

**How to cite this article**: Ratcliff, W. C. *et al*. Origins of multicellular evolvability in snowflake yeast. *Nat. Commun.* 6:6102 doi: 10.1038/ncomms7102 (2015).

## Supplementary Material

Supplementary Information Supplementary Methods

## Figures and Tables

**Table 1:** Top 10 downregulated genes.

**Gene**	**Fold change**	**FDR-adjusted** ***P*** **value**	**Function**
**SCW11**	−16.5	6.22·10^−12^	Cell wall protein with similarity to glucanases; may play a role in conjugation during mating based on its regulation by Ste12p.
**CTS1**	−13.6	2.86·10^−43^	Endochitinase, required for cell separation after mitosis; transcriptional activation during the G1 phase of the cell cycle is mediated by transcription factor Ace2p.
**DSE1**	−9.7	1.21·10^−12^	Daughter cell-specific protein, may regulate crosstalk between the mating and filamentation pathways; deletion affects cell separation after division and sensitivity to alpha-factor and drugs affecting the cell wall.
**AMN1**	−8.8	5.55·10^−9^	Protein required for daughter cell separation, multiple mitotic checkpoints, and chromosome stability; contains 12 degenerate leucin10-rich repeat motifs; expression is induced by the Mitotic Exit Network.
**DSE2**	−6	7.10·10^−20^	Daughter cell-specific secreted protein with similarity to glucanases, degrades cell wall from the daughter side causing daughter to separate from mother; expression is repressed by cAMP.
IZH4	−5.3	1.34·10^−7^	Membrane protein involved in zinc ion homeostasis, member of the four-protein IZH family, expression induced by fatty acids and altered zinc levels; deletion reduces sensitivity to excess zinc; possible role in sterol metabolism.
YNL277W-A	−4.9	0.013919	Putative protein of unknown function.
YFR057W	−4.7	0.040354	Putative protein of unknown function.
**DSE4**	−4.3	6.32·10^−08^	Daughter cell-specific secreted protein with similarity to glucanases, degrades cell wall from the daughter side causing daughter to separate from mother.
**SUN4**	−3.3	3.92·10^−05^	Cell wall protein related to glucanases, possibly involved in cell wall septation; member of the SUN family.

cAMP, cyclic AMP; FDR, false discovery rate.

Genes involved in daughter cell separation have been bolded. Gene function obtained from the *Saccharomyces* Genome Database.

**Table 2:** Locations of mutations in *ACE2* in five independently evolved lineages.

**Population**	**Codon position**	**Amino-acid change**
1	645	Val » Asp
2	645	Val » Asp
3	194	Gln » Stop
6	238	Gln » Stop
8	610	Cys » Ser

**Table 3:** Primers used in this study.

**Primer name**	**Strain (derivative of Y55)**	**Sequence (5′–3′)**	**Source**
ace2mx_F	*ace2::NATMX4*	CAAAGAAATCTATAGGACCAAAAACGGTGTTAATACAATCCGTACGCTGCAGGTCGAC	This study
ace2mx_R*	*ace2::NATMX4*	ATTATTTACTATGTTAATATCATGCATAGATAAATGTTCGATCGATGAATTCGAGCTCG	This study
ACE2wtmx_fusn_F	*ACE2-KANMX*	ACCAAAGGATGTGTGAAGCTGGTTTGTAGTAGTTAAAGGGATCGATGAATTCGAGCTCG	This study
ACEwtmx_fusn_R*	*ACE2-KANMX*	ATTTCTTTACGATTTACGTACACTGTAGTCTTAAGGGCCACGTACGCTGCAGGTCGAC	This study
ACE2wtmx_whole_F	*ace2/ace2::ACE2-KANMX*	CGTTGCAGGGAGACTCAA	This study
ACE2wtmx_whole_R*	*ace2/ace2::ACE2-KANMX*	TTAGGGTTATGTCCCTATAAACGATGACTATTGCCTTTTTATCGATGAATTCGAGCTCG	This study

^*^Reverse primer.
