# Supplementary material for: Origins of multicellular evolvability in snowflake yeast
Source: Nat Commun. 2015 Jan 20;6:6102. doi: 10.1038/ncomms7102 (PMC4309424; doi:10.1038/ncomms7102)
Supplement: Supplementary Information — Supplementary Methods [file ncomms7102-s1.pdf]

Supplementary Methods.

Derivation of the model predicting the distribution of cells in snowflake yeast clusters (Equation 1).

Assuming that the doubling time of each cell in the cluster is the same, we can create a model to predict the structure of the cluster by describing the number of cells of each distance (0, 1, 2, 3,…*x*) from the basal cell, given only the total number of cells in the cluster. Consider a single snowflake yeast cell (the 'basal cell'). At time zero we have a single cell distance 0 from the basal cell (the basal cell itself; Figure 2a). After one doubling, we have one cell distance 0, and one cell distance 1 from the basal cell (the basal cell's offspring). After another doubling each cell reproduces, resulting in one cell distance 0 from the basal cell, two cells that are distance 1, and one cell distance 2 from the basal cell (the basal cell's grandchild), *etc.*

We can describe the formation of a snowflake yeast cluster from a single cell mathematically with a series of recursions. Let $c_d(x)$ be the number of cells of distance *x* from the basal cell after *d* divisions (where $x \leq d$). The following relationship holds:

$$c_d(x) = \{c_{d-1}(x)\} + \{c_{d-1}(x-1)\}.$$

Using the same recursion again, this can be written as:

$$c_d(x) = \{c_{d-2}(x) + c_{d-2}(x-1)\} + \{c_{d-2}(x-1) + c_{d-2}(x-2)\}$$

which can be regrouped as:

$$c_d(x) = \{c_{d-2}(x)\} + 2\{c_{d-2}(x-1)\} + \{c_{d-2}(x-2)\}.$$

Using the same recursion again, it can be shown that:

$$c_d(x) = \{c_{d-3}(x)\} + 3\{c_{d-3}(x-1)\} + 3\{c_{d-3}(x-2)\} + \{c_{d-3}(x-3)\}.$$

With each added division *d,* we are simply moving down the rows of Pascal's triangle (Figure 2b). This can be rewritten as follows. Let $c_d(x)$ be the number of cells of distance *x* from the basal cell after *d* divisions (where $x \leq d$). Going back *d* cell divisions:

$$c_d(x) = \sum_{i=0}^{d} \binom{d}{i} c_0(x-i)$$

Given that there is only a single cell at the 0th cell division, it follows that:

$$c_0(x) = \begin{cases} 1 & if\ x = 0 \\ 0 & otherwise \end{cases}$$

Therefore, the only non-zero term in the above sum is when *i*=*x*, such that:

$$c_d(x) = \binom{d}{x} c_0(0) = \binom{d}{x} = \frac{d!}{x!\,(d-x)!}$$

If all cells are reproducing each time step, then after *d* divisions, the number of cells in the cluster, *h,* can be calculated as

$$h = \sum_{x=0}^{d} \binom{d}{x} = \sum_{x=0}^{d} \binom{d}{x} (1)^x (1)^{d-x} = (1+1)^d = 2^d$$

using the relation for a binomial expansion. The binomial expansion is appropriate, as the number of cells in the cluster doubles each division. Unfortunately, cellular reproduction within clusters is not actually synchronous as this model assumes. We therefore generalize the model to describe the branching pattern of clusters in which fraction *s* of the cells within the cluster have reproduced between two doublings. Let *h(t)* be the number of cells in the cluster at time *t,* and let the time points of doublings *d* and *d*+1 be *t* and *t'* respectively. Then the total number of cells that have reproduced between doublings *d* and *d* +1 is:

$$h(t') - h(t) = 2^{d+1} - 2^d = 2^d = h(t)$$

As a result,

$$s = \frac{h(t') - h(t)}{h(t)} = 1$$

Thus, when a full doubling has occurred, every cell has (on average) reproduced once. The parameter *s* will be less than unity when only some of the cells within the cluster have reproduced. For instance, if the cluster size is being measured from time point *t* to *t*+1, the following holds:

$$h(t+1) = h(t) + s(h(t))$$

Solving for *s* yields:

$$s = \frac{h(t+1) - h(t)}{h(t)}$$

If $d = \log_2(h(t))$, then

$$s = \frac{h(t+1) - 2^d}{2^d}$$

Here *s* is less than unity when *t*+1 occurs before the next doubling (*e.g.* time point *t*'). The distribution of cells (with respect to their distance from the basal cell) added to the cluster between doublings *d* and *d+1* is described by:

$$c_{d+1}(x) - c_d(x) = \binom{d+1}{x} - \binom{d}{x}$$

The expected branching pattern (number of cells of each distance from the basal cell) for clusters of any size can therefore be calculated as:

$$c_d(x) + s\left(\binom{d+1}{x} - \binom{d}{x}\right)$$
